# Supplementary material for: SH3Ps—Evolution and Diversity of a Family of Proteins Engaged in Plant Cytokinesis
Source: Int J Mol Sci. 2019 Nov 11;20(22):5623. doi: 10.3390/ijms20225623 (PMC6888108; doi:10.3390/ijms20225623)
Supplement: Supplementary file 1 [file ijms-20-05623-s001.zip › Supplements.zip!/Supplements_read_first.pdf]

Supplementary data for Baquero Forero and Cvrčková: SH3Ps – evolution and diversity of a family of proteins engaged in plant cytokinesis

The supplementary materials consist of the following:

Supplementary Figure S1: A maximum likelihood phylogenetic tree of seed plant SH3P2 protein sequences (in this file).

Supplementary Figure S2: Alignment of all full-length angiosperm SH3P sequences (in this file).

Supplementary Table S1: Inventory of SH3P homologs in selected plant species (in a separate MS Excel spreadsheet file TableS1.xls).

Supplementary Table S2: List of primers used for cloning into yeast two hybrid vectors (in this file).

Supplementary Table S3: List of primers used for cloning into Gateway™ compatible vectors.

Supplementary Data S1: Structural models of Arabidopsis SH3P1, SH3P2 and SH3P3 in the standard PDB format (in a separate Zip archive file DataS1.zip). Recommended viewer: DeepView (see Ref. [64], available at https://spdbv.vital-it.ch/.).

Supplementary Figure S1. A maximum likelihood phylogenetic tree of seed plant SH3P2 protein sequences. Symbols at branches denote bootstrap support (no symbols for support below 50 %), the arrow marks the position of the root of the angiosperm SH3P2 clade (with the gymnosperms serving as an outgroup). For species color-coding see Figure 1, for accession numbers of all sequences see Supplementary Table S1.

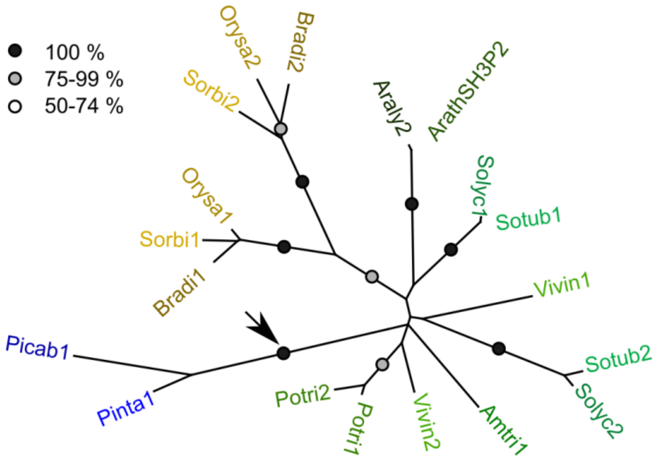

Supplementary Figure S2. Alignment of all full-length angiosperm SH3P sequences. Positions where amino acid category is conserved across all sequences are highlighted in green, those with amino acid category shared by all members of at least one clade and present in at least one member of another clade are shown on yellow background, clade-specific positions (i.e. those where amino acid type is shared by all members of one clade but not with other clades) are highlighted in turquoise. For amino acid categories definition see legend to Figure 3, for sequence description see Supplementary Table S1. Incomplete sequences or those lacking parts of well-conserved domains (possibly because of imperfect splicing prediction) have been omitted (*continued on next page*).

```
                   10        20        30        40        50        60        70        80
          ....|....|....|....|....|....|....|....|....|....|....|....|....|....|....|....|
ArathSH3P1 MEAIRKQAAKLREQVARQQQAVLKHL--GHV-NADAVVVDEEELHCHQKLQDLYSSTKAAKRLQRNIVRGLEGFIATGTK
Potri3     MEAIKKQATRLREQVAKQQQAVLKHL--GHF-SNEGIIVDEAELQCYQHLQNLYNSTRAAKHFQKNIVRGAEGFVSISSK
Potri4     MEAFKKQATRLREQVAKQQQAILKHL--GHL-SNEGIIVDEAELQCYQHLQNLYNSTRTAKHFQKNIVRGVEGFVSVSSK
Vivin3     MEAIRKQASKLREQVARQQQAVLKQL--GHF-GIETVVVDEAEQ---RQLQNLYNSTRTAKHFQKDIVRGIEGFVSTSSK
Solyc3     MEAIKKQATKLREQVAKQQQAILRQL--GHL-GHESVMVDEAELEIHQRLQELYMSTRAAKHFQRDIVRGVEGYISTSKK
Sotub3     MEAIKKQATKLREQVAKQQQAILRQL--GHL-GHESVMVDEAELEIHQRLQELYMSTRAAKHFQRDIVRGVEGYISTSKK
Amtri2     MDTLKRQASKFREQVSKQQQAVLRQF-STHG-GNDTVITDEAEVRCHKKLESLYISTRAAKHFQKDVVRSIEGLISTSSK
Bradi3     METLKKQASKLREHVAKQQQAVRKTF-STRY-NQDTSLVDEAELECHHNLQRLYNSTRAAKHFQRTIVRGVEGFVAVSTK
Sorbi3     MEALKKQASKLREHVAKQQQAVLKQF-SARY-NQDPSLVDEAELECHQNLQRLYSTTRAAKHFQRNIVRGVEGFIAISTK
Orysa3     METLRKQASKLREHVAKQQQAVRKQF-SARY-NQDPSLVDEAELECHQNLQRLYNSTRAAKHFQRSIVRGVEGFIAVSTK
ArathSH3P2 MDAIRKQASRLREQVARQQQAVFKQFGGGGY-GSG--LADEAELNQHQKLEKLYISTRAAKHYQRDIVRGVEGYIVTGSK
Amtri1     MDALRKQATKLREQVAKQQQAVFKQFGGGGYGGSDNVITDEAELQQHQKLEKLYISTRAGKHFQRDIVRGVEGFIVTGSK
Bradi1     MDAIRKQASKLREQVARQQQAVLKQF-GGGY-GADSVFADEGEAQQHTKLEKLYISTRAAKHFQRDIVRGVEGYIVTGSK
Bradi2     MEALWKQASRLKEQVAR--QGVFRQF-GGAYGNSDNAFTDESEVKLHQRLEKLYLSTRAAKHFQRDVVRGVEGYIVTGSK
Orysa1     MEAIRKQASKLREQVARQQQAVMKQF-GGGY-GADGAFADEAEAQQHSKLEKLYISTRAAKHFQRDIVRGVEGYIVTGSK
Orysa2     MEALWKQASRLKEQVSR--QGVFKPF-GAAYGNSENAFTDESEVNLHQRLEKLYLSTRAAKHFQRDIVRGVEGYIVTGSK
Potri1     MEAIRKQATKLREQVAKQQQAVLKQFGGGGYGGSDTLVTDEAELHQHQKLERLYISTRAGKHFQRDIVRGVEGYIVTGSK
Potri2     MEAIRKQATKLREQVAKQQQAVLKQFGAGGYGGSDTVITDEAELHQHQKLERLYISTRAGKHFQRDVVRGVEGYIVTGSK
Solyc1     MEAIRKQATRLREQVARQQQAVLKQFGAGGY-GSD-LVTDEAEMQQHHKLERLYISTRAAKHFQRDIVRGVEGYIVTGSK
Solyc2     MESIRKQATKLREQVAKQQQAVFKQF-SSGLGGPDNSVTDEVELQQHQKLEKLYISTRAGKHFQRDIIRGVEGYIITASK
Sotub1     MEAIRKQATRLREQVARQQQAVLKQFGAGGY-GSD-LVTDEAEMQQHHKLERLYISTRAAKHFQRDIVRGVEGYIVTGSK
Sotub2     MDSIRKQATKLREQVAKQQQAVFKQF-SSGLGGPDNSVTDEVELQQHQKLEKLYISTRAGKHFQRDIVRGVEGYIITASK
Sorbi1     MEAIRKQASKLREQVARQQQAVMKQF-GGGY-GADGVFADEAEAQQHSKLEKLYISTRAAKHFQRDIVRGVEGYIVTGSK
Sorbi2     MEALWKQASRLKDQVAR--QGVFKQF---GYGNSDNAFTDESEVKLHQRLEKLYLSTRAAKHFQRDIVRGVEGYIVTGSK
Vivin1     MEAIRKQATKIIDQVAKQQQAVLKQFSGGGYGGPDNVLTDESELQQHQKLEKLYISTRAGKHFQRDIVRGVEGFIVTGSK
Vivin2     MEAIRKQASKFREQVAKQQQAVLKQFGGGGYGGSDNVITDEAELQQHQKLEKLYISTRAGKHFQRDIVRGVEGFIVTGSK
ArathSH3P3 MDAFRRQASKLRDQVAKQQLAVIKQFSGTGYESSDVMVIDELEMQRHHQLDKLYRSTRSAKEFQRDIVKAAEAFTTIGLR
Bradi4     MEALRKQASKFKEQVAKQQQAVIKQFSTTGYERSDSVVIDEVELQQHQRLEKLYSSTRSGRDFQKDIVRAAEGFVSIGSK
Orysa4     MDVLRKQASKFKEQVAKQQQAVIKQFSTTGYEHSDAVVIDEVELQRHQQLEKLYTSTRSGRDFQKDIVRAAEGLVSIGIR
Potri5     MDALRKQASKLREQVAKQQQAVIKQFSSTGYESSDVMVIDEVEMHRHQQLEKMYSLTRAGKDFQKDILKAAESFTAIGYK
Solyc4     MDALRKQASKLREQVAKQQQAVIKQFSATGYESSDVMVIDEMEMQLHHQLDKLYKSTREKRDFQKEIVKAAETFTAIGYK
Sotub4     MDALRKQASKLREQVAKQQQAVIKQFSATGYESSDVMVIDEMEMQLHHQLDKLYKSTREKRDFQREIVKAAETFTAIGYK
Sorbi4     MEALRKQASKFKEQVAKQQQAVIKQFSTTGYERSDSVVIDEVELQRHQQLEKLYTSTRSGRDFQKDIVRAAEGLVSIGNK
Vivin4     MDALRKQASKFREQVAKQQQAVIKQFGGTGYESSDVMVIDEVEMQRHQQLEKLYRSTRAGKDFQKDIVRAGEAFTAIGYK

                   90       100       110       120       130       140       150       160
          ....|....|....|....|....|....|....|....|....|....|....|....|....|....|....|....|
ArathSH3P1 VVEIGLKFAEDFKKYGDENPDA-NTP-LSRVAHHFGTSYKSVEGERETLLGVLSEQVCEPIRTMIYSAPLEDARHLVNHY
Potri3     QMEILRKLADECCKYGAENQSE-NNY-VARTVLQFGASHNLMENEKEILLGVLNDQVSKPLRALITGAPLEDARHLTHRY
Potri4     QMEMLRRLADECCKYGAENQKE-NNY-VARAVLQFGASHNLMENEKETLVGVLNDQVSKPLRALITGAPLEDARRLTHHH
Vivin3     QMEIVRRMAEDCCKYGTENQST-GSP-LARAALYFGNSHSSMEKERETLLGVFCDQVSEPLRVLITGAPLEDARHLTHRY
Solyc3     QVEIARKLVDNCYKYGSEIQNG-PST-LPKVAVEFGTTHAAMEDQREIMLGVLGSQVCEPLRASIHGAPLEDARHLTHSY
Sotub3     QVEIARKLVDNCYKYGSEIQNG-PST-LPKVAVEFGTSHAAMEDQREIMLGVLGSQVCEPLRASIHGAPLEDARHLTHSY
Amtri2     QMEIATKLADDCCKYGNESHSS-NTT-LASASRQFGTSRNSMESERENLRRILSTQVSEPLRAMVMGAPLEDARLLTHRY
Bradi3     QMEIVKKLAEDCCKYGNNNQNL-GFV-LGIASVEFGKSHTQMEIEREKLLKVLGEQVFEPLREMIMSAPLEDARLLTYRY
Sorbi3     QMEIVKKLAEDCCKYGNDNQHF-GFA-LARASEEFGKSHKQIEKEREDLLKSLGEQVFEPLREMIMSAPLEDARLLTYRY
Orysa3     QMEIVKKLAEDCCRYGNDNQNF-GFI-LARASVEFGNSHSQMEKERENLLKFLGEQVFEPLREMIMSAPLEDARLLTYRY
ArathSH3P2 QVEIGTKLSEDSRKYGSENTCTNGNV-LTRAALNYGRARAQMEKERGNMLKALGTQVAEPLRAMVLGAPLEDARHLAQRY
Amtri1     QVEIGTKLSEDSRKYGNENSCTSGGT-LAKAALSYARGRAQIEKERGNLHKALGTQVAEPLRAMVLGAPLEDARHLAQRY
Bradi1     QVEIGNKLCEDGKKYGVENTCTSGST-LSRAALSFAKARSLMEKERGNLLKAFGTQVAEPLRAMVMGAPLEDARHLAQRY
Bradi2     QVEIGNKLCDDSQKYGIENTCTSGNT-LSKAATYYGKARSLIEKERGNMLKAFGTQVAEPLRAMVMGAPLEDARHLAQRY
Orysa1     QVEIGNKLCEDGKKYGAENTCTSGST-LSKAALCFAKARSLMEKERGNLLKALGTQVAEPLRAMVMGAPLEDARHLAQRY
Orysa2     QVDIGNKLSDDSQKYGTGNTCTSDNT-LSKAAMYYGKARSLMEKERGNMLRAFGTQVAEPLRAMVMGAPLEDARHLAQRY
Potri1     QVEIGTKFSEDSRKYGAENTCTSGNT-LSKAAVNYGRARAQMEKERGNLLKALGTQVAEPLRAMVMGAPLEDARHLAQRY
Potri2     QVEIGTRFSEDSRKYGAENTCTSGNT-LSKAAVNYANARAQMEKERGNLLKALGTQVAEPLRAMVMGAPLEDARHLAQRY
Solyc1     QVEIGTKLSEDSRKYGAENTCTSGTT-LSKAALGFSRARAQMEKERGNLLKALGTQVAEPLRAMVMGAPLEDARHLAQRY
Solyc2     QIEIGTRLSEDSRKYGAENKCTSGST-LSKAALSYSRAQGEIEKEREDLLKALGTQVAEPLRAMVVGAPLEDARHLAQRY
Sotub1     QVEIGTKLSEDSRKYGAENTCTSGTT-LSKAALGFSRARAQMEKERGNLLKALGTQVAEPLRAMVMGAPLEDARHLAQRY
Sotub2     QIEIGTRLSEDSRKYGAENTCTSGST-LSKAALSYSRAQGEIEKEREDLLKSLGTQVAEPLRAMVVGAPLEDARHLAQRY
Sorbi1     QVEIGNKLCEDGKKYGTENTCTSGST-LSKAALSFAKARSMMEKERGNLLKALGTQVAEPLRAMVMGAPLEDARHLAQRY
Sorbi2     QVEIGNKLSDDSQKYGVENTCTSGDT-LSKAATYFGKARSQMEKERGNMLKAFGTQVAEPLRAMVMGAPLEDARHLAQRY
Vivin1     QVEIGTKLSEDSRKYGVENTCTSGST-LSKAALSFARARAQMEKERGNLLKALGTKVGEPLRAMVMGAPLEDARHLAQRY
Vivin2     QVEIGTKLSEDCRKYGVENTCTSGNT-LSKAALNYGRARAQIEKERGNLLKALGTQVAEPLRAMVMGAPLEDARHLAQRY
ArathSH3P3 HIEAGTKLSEDCCRYGNENSQNIDENILAKAAAIYGDARKHVDKEQEDFNKLLASQVLDPLRAMVAGSPLEDARHLAQRY
Bradi4     HVEVGTKFSEDCFRYRGENNAD-EEA-LAKAASLYGGALRNVEKEYEDFSRTLSSQTIDPLRAMATCVPLEDARGLAQRY
Orysa4     HVEVGTKFSEDCYRYGGESSAS-DEA-LAKAASLYGGALRNVEKEYEEFNRILSSQTIDPLRAMAAGAPLEDARGLAQRY
Potri5     HVEAGTKLSEDCCRYGTENIN--ENI-LAKAAAIYGDAHKHVEQEQEDLNRLLFSQILDPLRAMITDSPLEDARHLAQRY
Solyc4     HIEAGTKLSEDCCKYGVENPN--DEV-LAKAASIYGDARKHAEKEVEDLNKLFFSQVLEPLRAMVAGSPLEDARHLAQRY
Sotub4     HIETGTKLSEDCCKYGVENPN--DEV-LAKAASIYGDARKHAEKEVEDLNKLFFSQVLEPMRAMVAGSPLEDARHLAQRY
Sorbi4     HIEVGTKFSEDCYRYGGENTAS-DEA-LGKAASLYGGALRNIEKEYEDFNRILSSQTIDPLRSMAMGAPLEDARGLAQRY
Vivin4     QIETGTKLSEDCCRYGAENIN--DNI-LARAASIYGDGLKHIAKEQEDLNKLLSSQILDPLRAMVTGAPLEDARHLAQRY
```

```
                    170       180       190       200       210       220       230       240
           ....|....|....|....|....|....|....|....|....|....|....|....|....|....|....|....|
ArathSH3P1 DRLRQEVEAQATDVLRRRSKLKESDISEEAYIKLKNSESRLAELKSSMKTLGKEATKAMLEVDDQQQNVTSQRLRALVEA
Potri3     DKLRQEVEAQAAEVLRRRSKTRDSEISAESCMKLQAAEARLTELKSTVMALGREATAAMSSVENQQQEITAQRLFSMVDA
Potri4     DKLRQAVEVQAAEVLRCRSKTRDSEISAESCTKLRAAEARLAELKSTVMSLGREATAAMSSVENQQQQITVQRLFSMIDA
Vivin3     ERLRQEVESQAADVLRRQAKFRDPATSAESSIKLQSAEAKLSELKSAMMALGREATAAMLSVEAQQQRITFQRLLTMVEA
Solyc3     DRMRQEFESQATEVIRRQSKFRE--ASTESLAKLKNAETRLSELKSSVLVLGKEATDAMLSVEEEQQQITFQKLLTMVDA
Sotub3     DRMRQEFESQATEVIRRQSKFRE--ASTESLAKLKNAETRLSELKSSVLVLGKEATDAMLSVEEEQQQISFQKLLTMVDA
Amtri2     DRIFQEVEAQVAEVGRRQLKAKEPGANADSAYKLQNAEMKLSELKSSLSALGKEATAAMMSVEAQQQRLTYQRLITMVEA
Bradi3     QRIRQDMESQIADVMRKQLKSKESSGNSD-SLKLQHAESKLSELRTTLAALGREATAAMEDVEAQQQQVTFDRLLAMVVA
Sorbi3     QRIRQDMESQIADVMRKQLKSKESSGNTDNSVKLQHAESKLSDLRTTLAALGREATAAMEAVEAQQQQVTYERLLAMVDA
Orysa3     QRIRQDMESQIADVMRKQLKSKESSGNADNSVKLQHAESKLSELRTTLSALGREATAAMEAVEVQQQQVTFDRLLAMVDA
ArathSH3P2 DRMRQEAEAQATEVARRQAKARESQGNPDILMKLESAEAKLHDLKSNMTILGKEAASALASVEDQQQKLTLERLLSMVES
Amtri1     DRMRQEAEAQAIEVSKRQMKVREGTGNVDNSAKLDAAEAKLHELKSNMAVLGKEAAAAMAAVEAQQQRLTLQRLIAMVES
Bradi1     DRMRQEAEAQAIEVSKRQMKLRETSGNGDMISRLEAAESKLQELKSNMGTLGKEAVAAMTAVEAQQQRLTLQRLIAMVES
Bradi2     DRMRQEADAQVVEVSRRQNRVRESAGNGEVISKLEAAEFKLEELKSSMVGLGKEAVAAMAAVEAQQQRLTLQRLIAMVEA
Orysa1     DRMRQEAEAQAIEVSKRQMKLRETSGNGDMISRLEAAESKLQELKSNMGVLGKEAVASMTAVEAQQQRLTLQRLIAMVES
Orysa2     DRMRQEAEAQAVEVSRRQNRVRESAPNGDVITKLEAAEYKLEELKSSMVGLGKEAVAAMAAVEAQQQRLTLQRLIAMVEA
Potri1     DRMRQEAEAQAIEVSKRQAKVREMPGSPELAMKLESAETKLQDLKSNMSILGKEAAAAMAAVEAQQQRLTLQRLIAMVEA
Potri2     DRMRQEAEAQAIEVAKRQAKVREMPGNSELAMKLESAETKLQDLKSNMAILGKEAAAAMAAVEAQQQRLTLQRLIAMVEA
Solyc1     DRMRQEAEAQAVEVSRRQAKLREGTGHPDMAYKLEAAETKLDDLKSNTNTLGKEAAAAMAAVEAQQQRLTLQRLIAMVES
Solyc2     DRVRQEAEAQAIEVSRRQAKVRESNGNPESVSKMEAAEAKLKDLKSNVATLGKEASTSMAAVEAQQQRLTLQRLIAMVEA
Sotub1     DRMRQEAEAQAVEVSRRQAKLREGTGHPDMAYKLEAAETKLDDLKSNTNTLGKEAAAAMAAVEAQQQRLTLQRLIAMVES
Sotub2     DRVRQEAEAQAIDISRRQAKVRESNGNPESVSKMEAAEAKLKDLKSNVATLGKEASTSMATVEAQQQRLTLQRLIAMVEA
Sorbi1     DRMRQEAEAQAIEVSKRQMKLREASGNSDMVARLEAAESKLQELKSNMGVLGKEAVAAMTAVEAQQQRLTLQRLIALVES
Sorbi2     DRMRQEAEAQVVEVSRRQNRVRESAGNGDMISKLEAAEYKLEELKSNMVGLGREAIAAMSAVETQQQRLTLQRLIALVEA
Vivin1     ERMRMEAEAQAIEVSKRQIKARESMGNPDNVLKLEAAEAKLHELKSNTAILGKEAVSAMAAVEGQQQRLTLQRIIAMVES
Vivin2     ERMRQEAEAQAIEVSKRQAKMREATGNADNTLKLEAAEAKLHDLKSNMAILGKEAAAAMAAVEAQQQRLTLQRLIATVES
ArathSH3P3 SRMRQEAETHATEVSRRQARVRE-APIPENVAKLQLAEAKMQELKANMAVLGKEATAALAAVESQQHRLTFQRLVAMVEG
Bradi4     SRMRHEAEILSAEIARRKARVRE-SPIAENTTKLQQSEAKMIEHKASMAVLGKEAVAALAAVESQQKRVTLQRLVGMVEA
Orysa4     SRMRHEAEILSAEIARRKQRVRE-APLAEHTTKLQQSESKMIEHKASMAVLGKEAAAALAAVESQQQRITLQRLVGMVEA
Potri5     SRMRQEAETQAAELSRRQARVRE-SPIPENIARLHAAEAKMQEIKANMAVLGKEAAAALAAVEAQQHRLTFQRLVAMVEG
Solyc4     SKMRQEAEIQATEVSRRQARVRE-APIPENVAKLHSAETKMQELKANMTVLGKEAAAALAAVESQQERLTFQRLVAMVEA
Sotub4     SKMRQEAEIQAAEVSRRQARVRE-APIPENVAKLHAAETKMQELKANMTILGKEAAAALAAVESQQERLTFQRLVAMVEA
Sorbi4     SRMRHEAEILSAEIARRKARVRE-APIPEHTTKLQQSEAKMIEHKASMAVLGKEAAAALAAVESQQQRVTLQRLVGVVEA
Vivin4     SRMRHEAETQAAEVSRRQARLRE-APNPENAARLNIAEAKMKELKANMAVLGKEAAAALAAVEAQQQRLSFQRLVAMVEG

                    250       260       270       280       290       300       310       320
           ....|....|....|....|....|....|....|....|....|....|....|....|....|....|....|....|
ArathSH3P1 ERSYHRNALDILDKLHSEMIAEEEAIGSSPKSLP-LHIEDSASLPQQEPNSNSSGEIKSNPLGKIKASRREEIKSNPQEV
Potri3     ERCYHQHVLTILDKLHAEMILEEQLNESALQSAT-TQRDV--ILPPEPKNNTSNGSENHMHPNHK---------------
Potri4     ERCYHQHVLTILDKLHAEMILEEQLNESSPQSET-TQREM--IVPSVHENNTSNGSKNHMDNNNQE--------------
Vivin3     ERSYHQTVLATLEKLYDEMIMEKKQNESSSQPIT-MEKDV--CVPTTSKDANSNGFDNHGHANQN---------------
Solyc3     ERSYHQNVVSILEKLHSEMLVEEQLSESPPQSSN---------SPRVLHDTTSNGTEHPETEDKS---------------
Sotub3     ERSYHQNVVSILEKLHSEMLVEEQLSESSPQSSN---------SPKVLHDTTSNGTEHPEAEDKS---------------
Amtri2     ERSYHKAVADNLDKLHAKMVVEKQRYESGALAD--VCL-----SSPSNKDKTENGSGGTANATQK---------------
Bradi3     ERTYHQNVADILNKLHDEMLHAKQHHGESDNHC--DEASS----EPSPEPKMSPTHEHSNS---------TSEDPALTET
Sorbi3     ERTYHQNAADILNKLHDEMLYAKHHNESANHYD--EQS-----SEPESDTGPAQVHSHSTS-----------EDPVLTKP
Orysa3     ERAYHQNAADILNKLHDEMVQAKHHDE-PENHY--DETSSDPKTAATHEHSRSTSEDHIFTNTSEPTRTETS-EPTRTET
ArathSH3P2 ERAYHQRVLQILDQLEGEMVSERQRIEAPSTPS--SADSM--PPPPSYEEANGVFASQM--HDTSTDS------------
Amtri1     ERTFHQRVLQILDQLEGDMASERHRIEASPNPV--LDN----PPPPSYEEANGLFTSQSPNYDVPSDS------------
Bradi1     ERSYHQRVLQILDQLEREMVSERQRIEGAPPPV--IESSM--PPPPSYEEVNGIFMRTPTVAELV-ET------------
Bradi2     ERTYHQKVLEILDHLEEEMVSERQKIEAPPTPA--AENYM--PPPPSYEEVNGMFASTS-TDQSV-NS------------
Orysa1     ERSYHQRVLQILDQLEREMVSERQRIEGAPPPA--VESSM--PPPPSYEEINGVFMRNPTVAELV-ET------------
Orysa2     ERAYHQRVLEILDHLEQEMVSERQKIEAPPTPS--AENYM-AQPPPSYDEVNGMFASSS-VDDSV-TS------------
Potri1     ERAYHQRVLQILDQLEGEMTSERQRIEAPPTPS--AENSM--PPPPSYEEVNGMYASQA-HNGTT-DS------------
Potri2     ERAYHQTVLQILDQLEGEMASERQRIEAPPTPS--AESSM--PPPPSYEEVNGVYASQAHHNGTT-DS------------
Solyc1     ERSYHQRILQILDQLEAEMLSERQRIEAAPAPP---LDTM--PPPPSYEEVNGVSTSPV-QNGST-DN------------
Solyc2     QRHYHERVLQILDQLEAEMMSERQRIEASPSSA--TDNTM--PPPPSYTDVNGGLHFKS-YDGSI-DG------------
Sotub1     ERSYHQRILQILDQLEAEMLSERQRIEAAPAPA-PPVDTM--PPPPSYEEVNGVSTSPV-QNGST-DN------------
Sotub2     QRHYHQRVLQILDQLEAEMMLERQRIEASPSSA--TDNSM--PPPPSYTEVNGGLHFKS-YDGFI-DG------------
Sorbi1     ERNYHQKVLQVLDQLEREMVFERQRIEGAPPPV--VESSM--PPPPAYEEVNGIFMRNT-VAELV-ET------------
Sorbi2     ERAYHKRVLEILDQLEQEMVSERQKIEAPPTPA--AENYMPPPPPPSYDEVNGAFASTS-VNESV-QS------------
Vivin1     ERSYHQSVLQILDQLESEMLAERQRIEAFPSPT--ANNAM--PPPPSYDEVNNVFASQT-YGEST-GS------------
Vivin2     ERAYHQRVLQILELLETEMISERQRIEAPPSPP--VENNT--PPPPSYEEVNGVFASQT-HNGSS-DS------------
ArathSH3P3 EKNYHLRIAAILSDIEAEMVTEKQHKESAPPAI---------PTENGSEK------------------------------
Bradi4     EKLFYLRLASILDDVEAEMSSEKQKRESAPPTI---------PSHKRAEK------------------------------
Orysa4     EKLFHLRLAAILDDVEAEMSSEKQKRESAPPTI---------HSHKRAEK------------------------------
Potri5     EKNYHLRIAAILSEVEAEMVSEKQQKESAPPVI--LLPVI--PSENGSEK------------------------------
Solyc4     EKLYHERVAVILGNIEAEIVSEKQRKEAAPPVA--TLPVN--PPAQIPEK------------------------------
Sotub4     EKLYHERVAVILGNIEAEIVSEKQRKEAAPPVA--TLAVN--PPAHIPEK------------------------------
Sorbi4     EKLFHLRLAAILDDVEAEMSSEKQRRESAPPII---------SLHKRAEK------------------------------
Vivin4     ERTYHQRIATIFGEVESEMVSEKQRKESAPPAI---------PSESHSEK------------------------------
```

```
                      330       340       350       360       370       380       390       400
            ....|....|....|....|....|....|....|....|....|....|....|....|....|....|....|....|
ArathSH3P1  TKPSPKDEMKSSPQEETKSNHQKEIKSSPQEEIKKSNGSDDHHNHQLLSQNDSYFLAKVVHPFDAQAPGELSLAVDDYVI
Potri3      ---------------------------------------------------DALYIAKVIHPFDAQAEGELSLFIDDFVV
Potri4      ---------------------------------------------------DGLFIAKVIHPFDAQAEGELSLSVDDFVV
Vivin3      ---------------------------------------------------GSYFIAKVIHPFDAQADGELGLSVDDYVV
Solyc3      ---------------------------------------------------PTYFIAKVMHSFDAQADGELSLEVGDYVV
Sotub3      ---------------------------------------------------PTYFIAKVMHSFDAQADGELSLEVGDYVV
Amtri2      ---------------------------------------------------AMYFLAEVLHPFDAQAEGELSLSVGDYVV
Bradi3      SEPTR-------------------------------------------NSQEVHYVGEVIHPFDAQADGELNISVGDYVV
Sorbi3      SESTG-------------------------------------------NSQEVQFLGEVIHPFDAQADGELSLAVGEYVV
Orysa3      SEPTR-------------------------------------------NGQEVHYVGEVIHPFDAQADGELSISVGDYVV
ArathSH3P2  ---------------------------------------------------MGYFLGEVLFPYHGVTDVELSLSTGEYVV
Amtri1      ---------------------------------------------------SSYFLGEVMHPYRAESDVELSLSVGDYVV
Bradi1      ---------------------------------------------------VEHFLAEAIQSYRAESETELNLSTGDYIV
Bradi2      ---------------------------------------------------VDFFLGEALDSFKAESESELNLSLGDIVI
Orysa1      ---------------------------------------------------VEFFLAEAIQSYRAESETELNLAAGDYIV
Orysa2      ---------------------------------------------------VDFFLGEALDSFKAESESELNLSAGDIVI
Potri1      ---------------------------------------------------ISYFLGEVMHSYQGQSDVELTLSIGDYVV
Potri2      ---------------------------------------------------MGYFLGEVMHSYQGQSDVELTLSIGDFVV
Solyc1      ---------------------------------------------------MGYFLGEVMYPYQAESDVELNLSVGEYIV
Solyc2      ---------------------------------------------------TTYYLAEVVHPYEAESDVELTLLIGDYVV
Sotub1      ---------------------------------------------------MGYFLGEVMYPYQAESDVELNLSVGEYIV
Sotub2      ---------------------------------------------------TTYFLAEVVHPYEAESDVELTLLIGDYVV
Sorbi1      ---------------------------------------------------VEYFLAEAIQSYRAESDTELNLSAGDYIV
Sorbi2      ---------------------------------------------------VDFFLGEALDSFKAESEFELTLSAGDIVI
Vivin1      ---------------------------------------------------MGYFLGEVMHSFQAESDVELNLSVGDHVV
Vivin2      ---------------------------------------------------ISYFLGEVMHSYQAESDVELNLSIGDFVV
ArathSH3P3  ---------------------------------------------------TSYFLAEVIHPFSAASEKELDLDKGDYIV
Bradi4      ---------------------------------------------------AQYFLAEAVNNFNGTTEKELSLIVGDYIV
Orysa4      ---------------------------------------------------AQYFLAEAVHNFNGTTEKELSLIVGDYVV
Potri5      ---------------------------------------------------TTYFLAEATHLFIAETEKELSLAVGDCIV
Solyc4      ---------------------------------------------------TKYFLAEAIHSFEAESEKELSLSVGDYVV
Sotub4      ---------------------------------------------------TKYFLAEAIHSFEAESEKELSLSVGDYVV
Sorbi4      ---------------------------------------------------AQYFLAEAMHNFNGTTEKELSLIVGDYVV
Vivin4      ---------------------------------------------------TMYFLAEAMHAFSAASEKELSLNVGDYVV

                      410       420       430       440       450
            ....|....|....|....|....|....|....|....|....|....|..
ArathSH3P1  VRQVAGTGWSEGEYKGKAGWFPSAYVEKQEKAPASKI-VESNSKQQ
Potri3      VRKVAPTGWSEGECKGKAGWFPSAYIEKHEKAPASKI-MEESSTP
Potri4      VRQVAPTGWSEGECKGKAGWFPSAYIEKHENSPERAT-SVSELLKAIPSGMV
Vivin3      VRQVAPNGWSEGECKGTAGWFPSAYVERRDKAPASVI-NEEASLAMIPN
Solyc3      VRQVAPNGWSEGECNGKSGWFPSAYAVMSDEVAASKM-VETDTTP
Sotub3      VRQVAPNGWSEGECNGTSGWFPSAYAVKSDEVAASKM-VETDTTP
Amtri2      VRQVSPAGWSEGECKGKAGWFPSAYIELRDKAPASKI-SDIDPQS
Bradi3      VRQVARNGWSEGECKGKAGWFPSAYVQQRDKAPASKV-IEPGLLTA
Sorbi3      VRQVAANGWSEGECKGKAGWFPSAYVEQRDKAPASKV-IEPGLLTT
Orysa3      VRQVAPNGWSEGECKGKAGWFPSAYVEQRDKAPASKV-IEPGLLTT
ArathSH3P2  VRKVTGSGWAEGECKGKAGWFPYGYIERRERVLASKV-SEVF
Amtri1      VRKVTRTGWAEGECKGKAGWFPSDYVERRERVLANKV-AEVF
Bradi1      VRKVSNNGWTEGECRGKAGWFPSEYIEKRDRVLASKV-AQVF
Bradi2      VRKVSSNGWAEGECKGKAGWFPHAYIERRERVLASKV-PHIF
Orysa1      VRKVSNNGWAEGECRGKAGWFPYDYIEKRDRVLASKV-AQVF
Orysa2      VRKISTNGWAEGECKGKAGWFPHGYIERRERVLASKV-PHIF
Potri1      VRKVTNNGWAEGECKGKAGWFPYGYIERRDRVLASKI-AEVF
Potri2      VRKVTNNGWAEGECKGKAGWFPYGYIERRDRVLASKI-AEVF
Solyc1      IRKVSNNGWAEGECKGRAGWFPFGYIERRDRVLASKV-AEVF
Solyc2      VRKVSNNGWAEGECKGKGGWFPFGYVERRERILATKV-AEVF
Sotub1      IRKVSNNGWAEGECKGRAGWFPFGYIERRDRVLASKV-AEVF
Sotub2      VRKVSNNGWAEGECKGKGGWFPFGYVERRERILATKV-AEVF
Sorbi1      VRKVSNNGWAEGECRGKAGWFPYDYIEKRERVLASKV-AQVF
Sorbi2      VRKISSNGWAEGECKGKAGWFPHAYIERQERVLASKV-PHIF
Vivin1      IRKVSNSGWAEGECRGKAGWFPVGYIERRERVLASKL-V
Vivin2      VRKVSNNGWAEGECKGKAGWFPFGYVEKRERVLASKM-AEVF
ArathSH3P3  VRKVSQTGWAEGECKGKAGWFPMAYIEKRQRLPTTNFAAEVY
Bradi4      VRQIAPNGWAEGECRGKAGWFPAGYVERRENIPPNKVFPQA
Orysa4      VRQIAPNGWAEGECKGVAGWFPAAYVERRENIPPNKVFPQA
Potri5      VRKVSPTGWSEGECKGKAGWFPSAYVEKRQRMSTSLA-AQAY
Solyc4      VRKVTQSGWSEGECQGRAGWFPSEYVEKRQRVPTSNGATEAY
Sotub4      VRKVTQSGWSEGECQGRAGWFPSEYVEKRQRVPTSNGATEVY
Sorbi4      VRQIAPNGWAEGECRGKAGWFPAAYVERRENIPPNKVFPQA
Vivin4      VRKVSHTGWSEGECKGKAGWFPSEYVAKRQRLPTSNVAAEVY
```

Supplementary Table S2: List of primers used for restriction enzyme-based cloning into yeast two hybrid vectors with restriction enzymes used and names indicating final destination vectors

Gene	Primer sequence 5´- 3´	Enzyme	Primer name
SH3P2	TTGGATCCGCAGAAACCCGCATCAACTC	BamHI	SH3P2_pGAD_for
	TTGAATTCTGTGAATAGCTCTTGATCTGGTGA	EcoRI	SH3P2_pGAD_rev
SH3P3	TTCTCGAGGTAGTTTGTTGGGGACAGGACA	XhoI	SH3P3_pGAD_for
	TTGAATTCGGGAAATCAAGAGGAGAAGTGAC	EcoRI	SH3P3_pGAD_rev
FH5	TTCATATGTACTCTGTTGGTTCCTCCATCAA	NdeI	FH5_pGBKT7_for
	TTCTGCAGAACTAGACTGATCCACGCGTCT	PstI	FH5_pGBKT7_rev
	TTCATATGTACTCTGTTGGTTCCTCCATCAA	NdeI	FH5_pGAD_for
	TTCCCGGGAACTAGACTGATCCACGCGTCT	XmaI	FH5_pGAD_rev

Supplementary Table S3: List of primers used for cloning into Gateway™ compatible vectors, PCR amplification of coding sequence with recombination sites and cloning into donor vectors for creating vectors for transient expression experiments. Primers M13for and M13rev were used for amplification of coding sequences of all three genes with recombination sites from pDONOR221 vectors for recombination into target vectors.

Gene	Primer sequence 5´- 3´	Vector (target)	Primer name
SH3P1	GGGGACAAGTTTGTACAAAAAAGCAGGCTTAATGGAAGCTATAAGAAAGCAAGCTG<br>GGGGACCACTTTGTACAAGAAAGCTGGGTACTGTTGCTTGGAGTTTGATTCCA	pDONOR221 (pGBKT7gw, pGADT7gw)	SH3P1 _ CDSgw for<br>SH3P1_ CDSgw rev
SH3P2	GGGGACAAGTTTGTACAAAAAAGCAGGCTTAATGGATGCAATTAGAAAACAAGC<br>GGGGACCACTTTGTACAAGAAAGCTGGGTATCAGAAAACTTCGGACACTTT	pDONOR221 (pGBKT7gw)	SH3P2 _for_gw<br>SH3P2_ rev_gw
SH3P3	GGGGACAAGTTTGTACAAAAAAGCAGGCTTAATGGATGCGTTTAGAAGACAAGC<br>GGGGACCACTTTGTACAAGAAAGCTGGGTATCAGTAAACTTCAGCAGCAAAGTT	pDONOR221 (pB7m34GW)	SH3P3 cds for gw<br>SH3P3 cds rev gw
FH5	GGGGACAAGTTTGTACAAAAAAGCAGGCTTAATGGTTGGAATGATTCGAGGAGG<br>GGGGACCACTTTGTACAAGAAAGCTGGGTAGTCTGAATCTGAACTAGACTGATCCAC	pDONOR221 (pB7m34GW)	FH5_for_gw<br>FH5_rev_gw
DRP1A	GGGGACAAGTTTGTACAAAAAAGCAGGCTTAATGGAAAATCTGATCTCTCTGGTT<br>GGGGACCACTTTGTACAAGAAAGCTGGGTATCACTTGGACCAAGCAACA	pDONOR221 (pGBKT7gw)	DRP1_for_gw<br>DRP1_rev_gw
All	GTAAAACGACGGCCAGT<br>AACAGCTATGACCAT	pGBKT7gw	M13for<br>M13rev
